# Current Status of Endolysin-Based Treatments against Gram-Negative Bacteria

**DOI:** 10.3390/antibiotics10101143

**Published:** 2021-09-22

**Authors:** Marco Túlio Pardini Gontijo, Genesy Perez Jorge, Marcelo Brocchi

**Affiliations:** Departamento de Genética, Evolução, Microbiologia e Imunologia, Instituto de Biologia, Universidade Estadual de Campinas (UNICAMP), Rua Monteiro Lobato 255, Campinas 13083-862, Brazil; g211546@dac.unicamp.br (G.P.J.); mbrocchi@unicamp.br (M.B.)

**Keywords:** nosocomial pathogen, bacteriophage, enzybiotic, peptide tag, bactericidal activity, biocontrol, antibiotic substitute

## Abstract

The prevalence of multidrug-resistant Gram-negative bacteria is a public health concern. Bacteriophages and bacteriophage-derived lytic enzymes have been studied in response to the emergence of multidrug-resistant bacteria. The availability of tRNAs and endolysin toxicity during recombinant protein expression is circumvented by codon optimization and lower expression levels using inducible pET-type plasmids and controlled cultivation conditions, respectively. The use of polyhistidine tags facilitates endolysin purification and alters antimicrobial activity. Outer membrane permeabilizers, such as organic acids, act synergistically with endolysins, but some endolysins permeate the outer membrane of Gram-negative bacteria *per se*. However, the outer membrane permeation mechanisms of endolysins remain unclear. Other strategies, such as the co-administration of endolysins with polymyxins, silver nanoparticles, and liposomes confer additional outer membrane permeation. Engineered endolysins comprising domains for outer membrane permeation is also a strategy used to overcome the current challenges on the control of multidrug-resistant Gram-negative bacteria. Metagenomics is a new strategy for screening endolysins with interesting antimicrobial properties from uncultured phage genomes. Here, we review the current state of the art on the heterologous expression of endolysin, showing the potential of bacteriophage endolysins in controlling bacterial infections.

## 1. Introduction

The indiscriminate use of antibiotics led to the emergence of antibiotic-resistant bacteria [1,2,3]. Antimicrobial resistance, the faster run of microbial evolution, and the failure in the development of antimicrobial drugs are health, economic, and social concerns [4,5]. Multidrug-resistant Gram-negative bacteria are a public health concern due to the low permeability of the outer membrane and the ease of exchange of antibiotic resistance genes [6]. Antimicrobial resistance in Gram-negative bacteria can arise from the mutation of genes carried in the chromosome or result from the acquisition, through horizontal gene transfer, of plasmids and transposons containing antibiotic resistance genes [7,8]. The main antimicrobial resistance mechanisms include antibiotic-inactivating enzymes, efflux pumps, and permeability or target modifications [9,10]. The most common multidrug-resistant Gram-negative bacteria are *Acinetobacter* spp., *Bordetella pertussis*, *Campylobacter* spp., *Citrobacter* spp., *Enterobacter* spp., *Escherichia coli*, *Klebsiella* spp., *Salmonella* spp., *Serratia marcescens*, *Shigella* spp., *Yersinia* spp., *Haemophilus influenzae*, *Helicobacter pylori*, *Legionella pneumophila*, *Neisseria* spp., *Pseudomonas aeruginosa*, and *Vibrio cholerae* [11].

Frederick Twort discovered bacteriophages (or phages) in 1915 [12]. Nonetheless, bacteriophages were first used in the biocontrol of bacteria by Felix d’Herelle [13]. Bacteriophages have been the study center of great scientific discoveries, such as gene regulation [14] and the CRISPR-Cas9 system [15], both Nobel-Prize-winning studies. The interest in bacteriophages has increased since the late 20th century as a response to the emergence of multidrug-resistant bacteria [16,17,18,19]. The lytic replication of bacteriophages requires the insertion of phage’s genetic material into the bacterial host, resulting in virion multiplication and, for most bacteriophages, the expression of endolysins, proteins that cause cell lysis throughout the disruption of peptidoglycan [20]. Cell lysis is caused by phage endolysins [21,22,23] and other accessory proteins such as holins and pinholins, that act in the cytoplasmic membrane [24,25,26], and spanins, that form junctions between the inner and outer membranes of Gram-negative bacteria [27].

**Figure 1:**
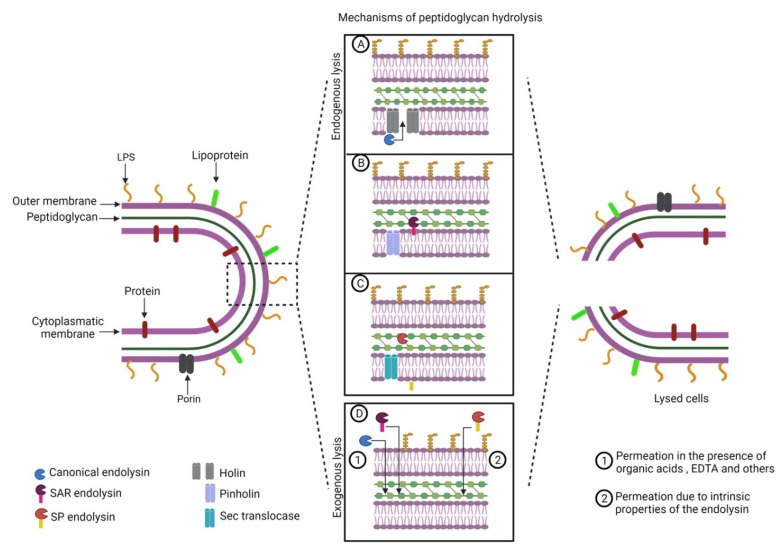
Mechanisms of peptidoglycan access of endolysins that act on Gram-negative bacteria: (**A**) endogenous lysis mediated by holins; (**B**) endogenous lysis mediated by pinholins; (**C**) endogenous lysis mediated by bacterial *Sec* translocases; (**D**) exogenous lysis.

Phage endolysins are subdivided into (I) glucosaminidases; (II) lysozymes or muramidases; (III) lytic transglycosylases; (IV) endopeptidases; and (V) amidases; depending on the peptidoglycan disruption mechanism [28,29,30]. The cleavage of a conserved structure such as the peptidoglycan points to endolysins as promising antimicrobials against multidrug-resistant Gram-negative bacteria. Bacteriophage endolysins are active from the inside of the cell. Bacteriophage-encoded accessory proteins help cytoplasmatic membrane export to reach the peptidoglycan [26,27]. Despite some advantages over the use of bacteriophages, such as broad-spectrum specificity and no report of bacterial resistance, the exogenous application of endolysins against Gram-negative bacteria is difficult due to the low permeability of the outer membrane [31] and some endolysins require additional strategies to permeate the outer membrane (Figure 1).

Several reviews approached bacterial therapy with bacteriophages [32,33,34,35]; however, the knowledge on the application of endolysins against Gram-negative bacteria is still scarce [29]. In this context, this review provides a comprehensive overview of the current status of research on endolysin-based treatments against Gram-negative bacteria, including expression vectors variety, cell systems used for protein expression, expression induction particularities, purification strategies, and antibacterial assay results. This review comprises a critical analysis of the most relevant papers in the last decade that focuses on endolysin heterologous expression.

## 2. Availability of tRNAs and Protein Toxicity Limit Endolysin Heterologous Expression

The heterologous expression of proteins finds two main difficulties that affect recombinant endolysin production: (I) the availability of tRNAs depending on the species in which the endolysin targets; and (II) protein toxicity [36]. Both of these situations result in little or no expression of the heterologous protein [37].

The availability of tRNAs, however, is circumvented by codon optimization strategies [38,39]. Most of the work on the heterologous expression of endolysins is conducted using the *E. coli* expression system (Table 1) and codon usage in this species is widely known [40,41]. Ph2119 endolysin, encoded by the *Thermus scotoductus* MAT2119 bacteriophage, possessed a significant number of rare codons for *E. coli*. Nevertheless, the codon-optimized gene was successfully introduced and expressed in *E. coli* and the purified enzyme showed high lytic activity toward Gram-positive bacteria and, to a lower degree, against Gram-negative pathogens such as *E. coli*, *S. marcescens*, *Pseudomonas fluorescens*, and *Salmonella enterica* [42]. The codon usage bias is more pronounced among Gram-negative and Gram-positive bacteria [43]. The codon preference and the abundance of tRNAs within any set of species is variable and codon–anticodon adaptation was reported in the relationship between phages and their host cells [44]. Closely related species, such as *E. coli* and *S. enterica*, have similar patterns of codon usage; however, different codons are preferred by *Helicobacter pylori*, also a Gram-negative bacterium [45]. These features highlight the requirement for codon optimization for a proper *E. coli* expression system even for endolysins encoded in the genomes of bacteriophages infecting closely related species.

**Figure 2:**
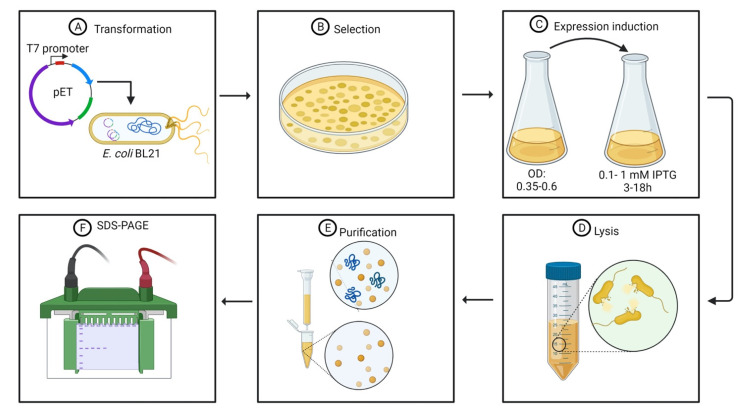
Standard protocol for endolysin heterologous expression: (**A**) bacterial transformation; (**B**) bacterial selection; (**C**) expression induction; (**D**) cell lysis; (**E**) endolysin purification; (**F**) SDS-PAGE.

Protein toxicity is an important feature of endolysins once the main goal of bacteriophage-derived lytic enzymes is an antibacterial activity. Toxic proteins, such as endolysins, affect the viability of the host cell during transformation and, in particular, during protein expression. Several authors have used pET plasmids (Table 1), which are regulated by the T7 bacteriophage promoter. The use of pET-type plasmids is advantageous due to: (I) high expression levels, resulting in proteins that can represent approximately 50% of the total protein content [37,41]; and (II) the maintenance of genes transcriptionally silent until induction [41,46]. The target endolysins are cloned in pET-type plasmids and transferred to *E. coli* strain (Figure 2A,B) comprising a chromosomal copy of the T7 RNA polymerase gene under the control of the lacUV5 promoter (*E. coli* strains BL21, BL21(DE3), BL21(DE3) pLysS, and others), which is induced in the presence of IsoPropyl-β-D-1-ThioGalactopyranoside (IPTG) [46]. Other levels of controlled expression are the decrease in the protein expression using lower temperatures and lower concentrations of the IPTG inducer (Figure 2C) [31,47,48,49].

Plotka et al. (2014) [42] observed that the overexpression of the synthetic gene of Ph2119 endolysin resulted in protein leakage. Nevertheless, decreasing incubation temperature to 30 °C was sufficient to reduce protein toxicity to *E. coli* cells and to decrease the inhibition of bacterial growth. Alternatively, several authors have used optimal *E. coli* growth temperatures (37 °C) and incubation times inferior to 5 h and obtained recombinant endolysins with promising antibacterial activity [50,51,52,53]. Antonova et al. (2019) [54] suggested little internal cell membrane penetration or insignificant lysis during protein expression.

## 3. Recombinant His-Tagged Endolysins Are Purified Using Affinity Chromatography

The first step for protein purification consists of cell lysis (Figure 2D). After incubation and consequent protein synthesis, the *E. coli* cells are collected by centrifugation, generally under refrigeration temperatures, at angular speeds superior to 6000× *g* for at least 10 min [48,54]. The harvested cells are then resuspended in a lysis buffer. The lysis buffer contains Tris HCl, NaCl with [54,55] or without [51] ethylenediaminetetraacetic acid (EDTA). Some authors have also associated Tris HCl and NaCl with ZnCl_2_ to enhance protein solubility [47]. Mixtures of NaH_2_PO_4_ and NaCl are also used as a lysis buffer [31,48,52]. Phenylmethylsulfonyl fluoride (PMSF) and other protease inhibitor cocktails can be added to the lysis buffer to prevent endolysin hydrolysis by nonspecific proteases present in the culture media [55].

Cell disruption is achieved by the addition of lysozyme followed by sonication [54,55], chloroform followed by shaking [47] and some authors have also caused lysis in *E. coli* cells using sonication [43,51,52,56]. Freeze-thawing cycles help cell lysis by sonication [31,48]. Cell debris is then removed by centrifugation, generally under refrigeration temperature to prevent protein degradation, at angular speeds superior to 10,000× *g* for at least 30 min [31,48,55], and the supernatant is filtered through a 0.22 μm pore size membrane [54,55]. The recombinant proteins present in the filtered supernatant are then subjected to purification.

The current state of the art concerning endolysin purification strategies lies in the use of polyhistidine tags, a standard procedure for general protein purification (Figure 2E). Histidine tags can be either added at the N-terminal [31,48,50,51] or at the C-terminal [47,52,54,55] region of the endolysin. Most of the endolysins evaluated are added to a 6-His fusion tag; however, 8- and 12-His tags have also been tested [55]. Several pET-type plasmids have an affinity tag composed of 6-His at the N-terminal region for further protein purification using metal-charged columns [46,57]. Recombinant proteins containing polyhistidine tags at the N-terminal region or comprising tags of more than six histidine residues must have their nucleotide sequence added on histidine codons [46,58]. The size and the position of the polyhistidine tag do not influence purification efficiency or wield. Nevertheless, polyhistidine tag size influences the antimicrobial activity of purified endolysins [55]. This influence will be discussed in Section 4.1.

The purification of endolysins possessing polyhistidine tags is made by affinity chromatography using nickel-charged columns employing a linear gradient of imidazole eluted in a solution containing Tris–HCl buffer and NaCl [31,47,48,50,51,52,54,55]. The polyhistidine tag interacts with the Ni^2+^ ion and, in the following step of elution, the imidazole ring, whose structure is similar to the side-chain of histidine residues, displaces the proteins during several passages of the elution buffer [59]. The following step consists of dialysis of the purified proteins against an elution buffer, such as PBS, to remove the imidazole excess [31,48]. Later, the purity of the proteins is assessed by SDS-PAGE (Figure 2F) [31,47,50,54,55,56] and the purified protein are stored at −80 °C in buffers either containing Tris–HCl, NaCl, and glycerol [50,51] or sodium phosphate, NaCl, and glycerol [52].

## 4. Endolysins as Therapeutic Agents against Gram-Negative Bacteria

Bacterial strains develop resistance to bacteriophages by mutations, receptor modification, passive adaptation, restriction modification, CRISPR-Cas, and pseudolysogeny [60]. However, there are no reports of bacteria developing resistance to endolysins [56]. A study performed by Briers et al. (2014) [61] evaluated the endolysin KZ144 fused with the antimicrobial peptide SMAP-29 and observed that the continuous exposure to the engineered endolysins did not lead to the development of antimicrobial resistance in *P. aeruginosa*. Similar results were found by other authors [62,63,64,65]. Resistance to antibiotics occurs because generally antibiotics act on inhibiting essential metabolic pathways of bacteria leading to cell death. However, bacteria find alternative pathways to overcome antimicrobial exposure [66]. Once endolysins act on such a conserved structure as the peptidoglycan, it is difficult for bacteria to evolve means of resistance to endolysins without damaging cell integrity [67,68].

The term “enzybiotic” was attributed in 2001 to define the application of bacteriophage-derived enzymes for treating bacterial infections [69]. In comparison to the use of bacteriophages, the application of endolysins as antibacterial agents has several advantages [70]: (I) endolysins have broad-spectrum antimicrobial activity, which can also be a disadvantage by disturbing the microbiota; however, (II) antimicrobial potential can be modified by changing the endolysin concentration; (III) endolysins can act on both dormant and growing cells; (IV) no bacterial resistance to endolysins has been reported; (V) action against bacterial biofilms; (VI) lower degree of antibody neutralization; (VII) better-defined pharmacokinetics; and (VIII) versatility on combined application with organic acids, antibiotics, proteins, and other active molecules.

Table 2 summarizes some studies that have explored the application of recombinant endolysins against Gram-negative pathogens.

### 4.1. Some Native Endolysins Reach the Peptidoglycan Layer Per Se

Antonova et al. (2019) [54] obtained a minimal active concentration of 0.5 μg·mL^−1^ for LysAm24, encoded by the *Acinetobacter* phage AM24, and LysECD7, encoded by the *Escherichia* phage ECD7, and 0.5 μg·mL^−1^ for LysSi3, encoded by the *Enterobacteria* phage UAB_Phi87, recombinant endolysins, reducing up to 3-log cycles of *Acinetobacter baumannii* counts. Endolysin concentrations superior to 5 μg·mL^−1^ (LysAm24 and LysECD7) and 50 μg·mL^−1^ (LysSi3) eliminated *A. baumannii* growth without the addition of outer membrane permeabilizers. The authors hypothesized that the cell permeation of recombinant endolysins was improved by the 8-His tag at the C-terminal region. The authors also observed that endolysins were more active when applied in the bacterial exponential growth phase than in the stationary phase.

In the following study, Antonova et al. (2020) [55] evaluated the polyhistidine tag hypothesis raised in their previous study. The author evaluated the antimicrobial activity of LysECD7 recombinant endolysin (native LysECD7, LysECD7-6his, LysECD7-8his, LysECD7-12his) at 1.0 μg·mL^−1^ over a pH gradient. All His-tagged endolysins were less active compared to the native enzyme. The authors observed that the outer membrane permeation is achieved by other features other than the polyhistidine tags.

Lim et al. (2014) [52] observed a lytic activity of the SPN9CC endolysin, encoded by the *Salmonella* bacteriophage SPN9CC, against *E. coli* at an endolysin concentration of 300 μg·mL^−1^ after 1 h. The authors suggested that the presence of a transmembrane helix in the N-terminal region of the endolysin comprising a signal–arrest–release (SAR) domain could be responsible for the outer membrane penetration. The study observed that the SAR domain plays a major role in the antibacterial activity; once SPN9CC endolysin deleted of some amino acids in the N-terminal region, it showed no lytic activity.

Guo et al. (2017) [24] observed that LysPA26 recombinant endolysin, predicted to belong to the lysozyme-like domain family and encoded by the *Pseudomonas* bacteriophage JD010, showed bactericidal activity against exponentially growing *P. aeruginosa* as a function of concentration, reaching a maximum activity at 500 μg·mL^−1^ without outer membrane permeabilizers. The concentration of 500 μg·mL^−1^ reduced 100% of the *P. aeruginosa* cells, compared to a reduction of 20% at a 50 μg·mL^−1^. Additionally, *P. aeruginosa* biofilm was significantly reduced after the addition of LysPA26 up to 50 μg.

A study conducted by Kim et al. (2020) [47] showed that LysSS recombinant endolysin, belonging to the lyzozyme family and encoded by the *Salmonella* bacteriophage SS3e, had antibacterial activity against *A.baumannii*, *E. coli*, *Klebsiella pneumoniae*, *P. aeruginosa*, and *Salmonella* without the addition of outer permeabilizers. The minimum inhibitory concentration was inferior to 750 μg·mL^−1^ and the minimum bactericidal concentration for *A. baumannii* was 250 μg·mL^−1^ and 500 μg·mL^−1^ for *P. aeruginosa*. LySS endolysin showed antimicrobial activity against Gram-positive pathogen *S. aureus* (750 μg·mL^−1^); such activity against both Gram-positive and Gram-negative bacteria is unusual. The in vivo mice model with an intraperitoneally induced *A. baumannii* infection treated with 125 μg of LysSS resulted in a 40% survival rate after 4 days of infection.

The mechanisms of outer membrane permeation of the endolysins evaluated by Guo et al. (2017) and Kim et al. (2020) remain unclear.

### 4.2. Outer Membrane Permeabilizers Improve Native Endolysin Diffusion

Although some endolysins act exogenously against Gram-negative bacteria in the absence of outer membrane permeabilizers, the antibacterial activity of endolysins is enhanced when combined with chemical permeabilizers [24,52,54]. Some studies mentioned in Section 4.1 observed better antibacterial activity combining purified endolysins with outer membrane permeabilizers. Antonova et al. (2019) [54] observed that the addition of EDTA improved the endolysin antibacterial activity in the stationary phage, inhibiting *P. aeruginosa*, *A. baumannii*, and *K. pneumoniae*.

Guo et al. (2017) [24] observed that 1 mM EDTA enhanced the LysPA26 antimicrobial activity and Lim et al. (2014) [52] observed that the tested Gram-negative bacteria (*S. enterica*, *E. coli*, *P. aeruginosa*, *Pseudomonas putida*, *Shigella boydii*, *Shigella flexneri*, *Vibrio fischeri*, and *Vibrio vulnificus*) were more susceptible to SPN9CC endolysin (0.5 μg·mL^−1^) after an EDTA pretreatment.

Other studies, however, reported endolysins functioning only in the presence of outer membrane permeabilizers. Oliveira et al. (2014) [48] observed that in the presence of organic acids (0.5 mM EDTA, 2 mM of citric acid, or 5 mM of malic acid), antibacterial activity was observed for Lys68, encoded by the *Salmonella* phage phi68. Higher log reduction values were obtained using malic acid, acting against *Salmonella* Typhimurium, *A. baumannii*, *P. aeruginosa*, *Pseudomonas fluorescens*, *Shigella sonnei*, *E. coli*, *Cronobacter sakazakii*, *Pantoea agglomerans*, *Enterobacter amnigenus*, *Proteus mirabilis*, and *Salmonella bongori*. A similar study revealed that the pretreatment of *E. coli* with EDTA (0.50 mM), citric acid (0.36 mM), malic acid (0.60 mM), lactic acid (1.20 mM), benzoic acid (1.20 mM), or acetic acid (1.20 mM) enhances the antimicrobial activity of ABgp46 endolysin, encoded by the *Acinetobacter* phage vb_AbaP_CEB1 [31].

Lim et al. (2012) [51] treated *E. coli* cells with 0.03 μg·mL^−1^ of SPN1S endolysin, encoded by the *Salmonella* bacteriophage SPN1S, and 5 mM EDTA, resulting in an approximate 2-log reduction after 2 h and a 4-log reduction using 0.100 μg·mL^−1^ endolysin and 10 mM EDTA. All tested Gram-negative bacteria (*E. coli*, *Salmonella* Typhimurium, *Salmonella* Typhi, *Salmonella* Paratyphi, *S. flexneri*, *P. aeruginosa*, *P. putida*, *C. sakazakii*, and *V. vulnificus*) were lysed by SPN1S endolysin at 0.05 μg·mL^−1^ after 10 min, however, no activity was detected against Gram-positive bacteria.

Bai et al. (2019) [50] purified the BSP16Lys endolysin, encoded by the *Salmonella* bacteriophage BSP16, and observed antimicrobial activity against EDTA pretreated *Salmonella* Typhimurium and *E. coli* cells at a concentration of 0.15 μM. However, no activity was observed against EDTA non-treated cells. The absence of antimicrobial activity of BSP16Lys in the absence of EDTA was bypassed after endolysin encapsulation in liposomes. Encapsulated endolysins were evaluated against *Salmonella* Typhimurium and *E. coli* cells without an EDTA pretreatment and showed similar results to the free BSP16Lys applied in EDTA treated cells.

Blasco et al. (2020) [56] evaluated the combined effect of endolysins and the membrane-destabilizing antibiotic colistin. The combination of the endolysin ElyA1, encoded by the *Acinetobacter* bacteriophage Ab105-1phi, with colistin reduced the minimal inhibitory concentration of colistin by up to ¼, resulting in a 2-log reduction in the counts of both *A. baumannii* and *P. aeruginosa* after 6 h. The in vivo survival model of Galleria mellonella-infected larvae treated with colistin (¼ MIC) combined with ElyA1 endolysin (25 μg·mL^−1^) was higher than that treated only with colistin (¼ MIC). In mice, superficial skin wounds infected with *A. baumannii* treated with colistin combined with 50 μg and 350 μg of ElyAl showed cell counts lower in the colistin combination treatments than in the buffer control.

Ciepluch et al. (2019) [71] combined bacteriophage lytic enzymes with dendritic silver nanoparticles (AgNPs), considered a potent outer membrane-disrupting agent. It was shown that dendritic AgNPs create a complex with lipopolysaccharides (LPSs) comprised in the outer membrane of Gram-negative bacteria and enhanced the antibacterial effect of the endolysins evaluated. A concentration of 20 μg·mL^−1^ of dendritic AgNPs with endolysin (5 μM) reduced up to 80% in the OD600 of the *P. aeruginosa* PAO1 model compared to the non-treated bacterial culture.

The use of outer membrane permeabilizers, such as EDTA [72] and antibiotics [73] leading to synergistic drug interactions, and liposomes [74] for assisted drug delivery have promising clinical impact. Further studies are still required to determine the proper outer membrane permeabilizers for further in vivo investigation.

### 4.3. Protein Design Favors Endolysin Permeation through the Outer Membrane

The limited application of endolysins against Gram-negative bacteria due to the low permeability of the outer membrane resulted in the development of engineered endolysins, including (I) innolysins; (II) lysocins; and (II) artilysins.

Innolysins combine the peptidoglycan disruption activity of native endolysins with phage receptor binding proteins, adhesion structural proteins that bind to surface receptors found in the outer membrane of Gram-negative bacteria, such as proteins, LPS, capsule components, pili, and flagella [75]. Nine of the twelve innolysins constructed by Zampara et al. (2020) [75] presented muralytic activity against *P. aeruginosa* PAO1, ranging from 126 to 771 μmol∙min^−1^∙mL^−1^. A study by Lukacik et al. (2012) [76] constructed an endolysin hybrid containing the T4 lysozyme attached to the FyuA-targeting domain found in pesticin. The endolysin hybrid could kill model *E. coli* cells expressing the FyuA gene and other species of bacteria encoding the FyuA gene, such as *Yersinia pseudotuberculosis* and *Y. pestis*. Additionally, Briers et al. (2014) [77] developed the concept of artilysins based on the construction of endolysin hybrids containing LPS-destabilizing peptides (cationic, hydrophobic, or amphipathic domains). Artilysins could kill multidrug-resistant bacteria such as *P. aeruginosa*, *A. baumannii*, *E. coli*, and *Salmonella* Typhimurium in vitro and in vivo (*Caenorhabditis elegans* and keratinocytes).

## 5. Thermally Resistant Endolysins Are Promising Candidates for Drug Development

Thermally resistant endolysins are important in terms of clinical applications; once thermostable proteins are more easily handled and tend to keep their activity for longer periods. Mikoulinskaia et al. (2018) [78] identified, cloned, and performed the biochemical characterization of two novel thermally resistant endolysins: RB43 endolysin retained 81% of its activity after a 10 min at 90 °C and RB49 endolysin that retained 27% of its activity after the same treatment. Plotka et al. (2014) [42], on the other hand, evaluated the Ph2119 endolysin, whose activity remained around 87% after 6 h at 95 °C and optimum temperature ranges from 50 °C to 78 °C. The endolysin showed lytic activity toward Gram-positive thermophiles (*Thermus scotoductus*, *Thermus thermophilus*, and *Thermus flavus*), and Gram-negative bacteria (*E. coli*, *S. marcescens*, *P. fluorescens*, and *S. enterica*). Plotka et al. (2015) [79] obtained similar results for Ts2631 endolysin, whose activity was retained at 64.8% after 2 h at 95 °C and with antibacterial spectrum including Gram-positive thermophiles and Gram-negative bacteria. Plotka et al. (2019) [80] completed the study with Ts2631 endolysin and evaluated the combined effect of the enzyme with EDTA and organic acids. The results revealed at least a 2.9-log reduction in the counts of carbapenem-resistant *A. baumannii* and 6-log reductions in the counts of multidrug-resistant *Citrobacter braakii*. Finally, Wang et al. (2020) [81] evaluated MMPphg thermally stable endolysin (80% of its activity retained after 30 min at 65 °C) and observed antimicrobial activity against both Gram-negative and Gram-positive antibiotic-resistant strains, such as *E. coli* O157:H7, *S. aureus*, and *K. pneumonia*.

## 6. Bacteriophage Depolymerases Might Act Synergistically with Endolysins

Depolymerases are phage-derived enzymes that degrade capsular polysaccharides, extracellular polymeric substances, and the O-antigen, offering a promising tool for controlling multidrug-resistant bacteria [82]. Wang et al. (2020) [83] evaluated the action of the purified depolymerase Dp49 (250 μg·mL^−1^), encoded by the *Acinetobacter* phage vB_AbaM_IME285, combined with activated serum and observed that the bacterial counts decreased, reaching up to a 4-log reduction. All mice evaluated infected with the lethal dose of *A. baumannii* (200 μL at 6 × 10^7^ CFU) died in the first 24 h. However, all mice treated with Dp49 (50 μg) survived. Similar results were found by Pan et al. (2015) [84] evaluating the activity of depolymerases against carbapenem-resistant *K. pneumoniae* strains. Studies accounting for the combined effect of depolymerases and phage endolysins on Gram-negative bacteria are still missing.

## 7. Metagenomics in the Discovery of New Endolysins

Metagenomics could be used as a strategy to screen potential endolysins for potential in vitro and in vivo studies. Fernández-Ruiz et al. (2018) [85] used a bioinformatics approach to analyze the genomes of approximately 200 thousand uncultured bacteriophages. The authors discovered 2628 putative endolysins, including endolysins comprising novel domain architectures. Currently, there is a growing body of research evaluating the distribution of unculturable viral genomes across the globe. Metagenomics will generate an abundance of genetic information from uncultured bacteriophages, and this massive data will potentially expand the knowledge on endolysin diversity and evolution. Therefore, further studies require the determination of biodiversity-shaping factors that affect the bacteriophage–endolysin–bacteria ecology.

## 8. Limitations of Endolysin Therapy

Despite promising results and high therapeutic potential, there are four major limitations on endolysin-based treatment: (I) pharmacokinetics and immunomodulatory aspects; (II) drug delivery methods variety; (III) specificity for general treatments; and (IV) regulatory issues.

First, defining endolysin pharmacokinetics and immunomodulatory properties currently comprises a clinical gap. These characteristics were explored in Section 4. Multimodular proteins, proteins with different catalytic and cell-binding domains, lead to considerable variations in antimicrobial activity and will probably affect formulation design and pharmacokinetics [86]. These characteristics point to the idea that endolysin systems need to be carefully designed for each endolysin.

The majority of endolysin-based products have topical uses, which are ideal for skin and wound infections, but not suitable for many other parts of the body. Due to the protein character of endolysins, the oral administration of endolysins is difficult given that drugs must transit the entirety of the gastrointestinal tract [87]. Considering other administration routes, endolysins can trigger the immune system [88]. This constraint highlights the need for investigation to protect endolysins from the immune system.

In addition, Murray et al. (2021) [89] highlighted that endolysins generally have more specificity than common antibiotics. Most of the studies on endolysins are conducted on specific infections. In contrast, many antibiotics are prescribed and used by people with simple illnesses. The main challenge is to determine whether such personalized treatment is an economically viable alternative.

Concerning regulatory issues, most of the studies on endolysins-based treatments are based on recombinant DNA technology and heterologous expression. This method has specific requirements and guidelines that make endolysin production and use in human therapy difficult [89] and the legal framework for endolysin applications is currently unstandardized [90].

Considering these four limitations, especially the individuality of endolysin treatments and scale-up efforts, the cost of the development of endolysin-based drugs is currently higher than that of conventional antibiotics [86]. Despite these limitations, endolysins-based treatments are expected to enter clinical trials in a near future.

## 9. Conclusions

The antimicrobial resistance crisis has boosted endolysin research in the past few years, and endolysins show great potential to replace or supplement antibiotic treatments. There is no complexity in terms of the expression vector and expression system in the heterologous expression of endolysins, and purification strategies for recombinant endolysins are well established. Nevertheless, extensive knowledge on both efficacity and size of the His tag is still required to determine the most effective antibacterial effect of endolysins against Gram-negative bacteria. Recombinant endolysins act on the Gram-negative cells either in the presence or in the absence of outer membrane permeabilizers. However, there are still many challenges to be addressed before the clinical use of endolysins, including the administration mechanisms of endolysin-based drugs. As previously addressed by Love et al. (2018) [91], the current status of endolysin-based treatments against Gram-negative pathogens highlights the study of scale-up production and the definition of regulations in several fields of application.

## Figures and Tables

**Table 1:** Heterologous expression vector, system, and induction of endolysins against Gram-negative bacteria.

Endolysin	Vector	System	Induction	Reference
LysAm24	pET42b	BL21(DE3) pLysS	LB broth (37 °C, 240 rpm) to an OD600 of 0.55–0.65 and 1 mM IPTG at 37 °C for 3 h	[54]
LysECD7				
LysSi3				
BSP16Lys	pET28a	BL21(DE3)	LB broth (37 °C) to an OD600 of 0.5 and 0.5 mM IPTG at 37 °C for 3 h	[55]
LysAB54	pET28a	BL21(DE3)	LB broth (37 °C) to an OD600 of 0.6 and 1.0 mM IPTG at 16 °C for 16 h	[49]
ElyA1	pET28a	Rosetta (DE3) pLysS	LB broth (37 °C, 180 rpm) and 1 mM IPTG at 30 °C for 5 h	[56]
ElyA2				
LysPA26	pET28b	BL21(DE3)	LB broth (37 °C) and 1 mM IPTG at 25 °C for 5 h	[24]
LysSS	pET21	BL21(DE3)	LB broth (37 °C) to an OD600 of 0.5 and 0.1 mM IPTG at 18 °C for 16 h	[47]
LysSPN1S	pET15	BL21(DE3)	LB broth to an OD600 of 0.6 and 1 mM IPTG for 3 h	[51]
LysSPN9CC	pET29b	BL21(DE3)	LB broth to an OD600 of 0.6 and 1 mM IPTG for 4 h	[52]
Lys68	pET28a	BL21(DE3)	LB broth (37 °C, 120 rpm) to an OD600 of 0.6 and 0.5 mM IPTG at 16 °C for 18 h	[48]
Abgp46	pET15b	BL21(DE3)		[31]
Lysep3	pET28a	BL21(DE3)	LB broth (37 °C, 180 rpm) to an OD600 of 0.35 and 0.1 mM IPTG at 37 °C for 3 h	[53]

**Table 2:** Summary of research that evaluated the exogenous antimicrobial activity of endolysins against Gram-negative pathogens ^1^.

Type of Study	Main Findings
Native endolysins	Polyhistidine tags influence outer membrane permeation. Transmembrane regions in the N-terminal region of endolysins comprising signal–arrest–release domains could be responsible for outer membrane permeation. The mechanisms of outer membrane permeation by endolysins remain inconclusive, but both in vitro and in vivo research revealed that some native endolysins can inhibit Gram-negative bacteria.
Native endolysins combined with outer membrane permeabilizers	Organic acids improve the antimicrobial activity of endolysins or facilitate the permeation of endolysins. Liposomes, silver nanoparticles, and polymyxins facilitate outer membrane permeation of endolysins.
Engineered endolysins	Hybrids of endolysins with outer membrane receptors, bacteriocins, and outer membrane-destabilizing peptides enhance endolysin diffusion.

^1^ Check text for references.

## Data Availability

Not applicable.

## References

[1] Aslam B., Wang W., Arshad M.I., Khurshid M., Muzammil S., Rasool M.H., Nisar M.A., Alvi R.F., Aslam M.A., Qamar M.U. (2018). Antibiotic resistance: A rundown of a global crisis. Infect. Drug Resist..

[2] Ventola C.L. (2015). The Antibiotic Resistance Crisis: Part 1: Causes and threats. Pharm. Ther..

[3] Boggione D.M., Batalha L.S., Gontijo M.T., Lopez M.E., Teixeira A.V., Santos I.J., Mendonça R.C. (2017). Evaluation of microencapsulation of the UFV-AREG1 bacteriophage in alginate-Ca microcapsules using microfluidic devices. Colloids Surfaces B Biointerfaces.

[4] Gould K. (2016). Antibiotics: From prehistory to the present day. J. Antimicrob. Chemother..

[5] Pepper J.W. (2014). The evolution of bacterial social life: From the ivory tower to the front lines of public health. Evol. Med. Public Health.

[6] Zgurskaya H.I., López C.A., Gnanakaran S. (2015). Permeability Barrier of Gram-Negative Cell Envelopes and Approaches to Bypass It. ACS Infect. Dis..

[7] Bello A., Dingle T. (2018). What’s That Resistance Mechanism? Understanding Genetic Determinants of Gram-Negative Bacterial Resistance. Clin. Microbiol. Newsl..

[8] Eichenberger E., Thaden J.T. (2019). Epidemiology and Mechanisms of Resistance of Extensively Drug Resistant Gram-Negative Bacteria. Antibiotics.

[9] Breijyeh Z., Jubeh B., Karaman R. (2020). Resistance of Gram-Negative Bacteria to Current Antibacterial Agents and Approaches to Resolve It. Molecules.

[10] Étienne R., Woerther P.-L., Barbier F. (2015). Mechanisms of antimicrobial resistance in Gram-negative bacilli. Ann. Intensiv. Care.

[11] Exner M., Bhattacharya S., Christiansen B., Gebel J., Goroncy-Bermes P., Hartemann P., Heeg P., Ilschner C., Kramer A., Larson E. (2017). Antibiotic resistance: What is so special about multidrug-resistant Gram-negative bacteria?. GMS Hyg. Infect. Control.

[12] Twort F. (1915). An Investigation on the Nature of Ultra-Microscopic Viruses. Lancet.

[13] D’Herelle F. (1931). Bacteriophage as a Treatment in Acute Medical and Surgical Infections. Bull. N. Y. Acad. Med..

[14] Jacob F., Monod J. (1961). Genetic regulatory mechanisms in the synthesis of proteins. J. Mol. Biol..

[15] Ishino Y., Krupovic M., Forterre P. (2018). History of CRISPR-Cas from Encounter with a Mysterious Repeated Sequence to Genome Editing Technology. J. Bacteriol..

[16] Lopez M.E.S., Batalha L.S., Vidigal P.M.P., Albino L.A.A., Boggione D.M.G., Gontijo M.T.P., Bazzolli D.M.S., Mendonca R.C.S. (2016). Genome Sequence of the Enterohemorrhagic Escherichia coli Bacteriophage UFV-AREG1. Genome Announc..

[17] Gontijo M.T., Batalha L.S., Lopez M.E., Albino L.A. (2017). Bacteriophage Genome Sequencing: A New Alternative to Understand Biochemical Interactions between Prokaryotic Cells and Phages. J. Microb. Biochem. Technol..

[18] Lopez M., Gontijo M., Batalha L., Mendonca R. (2018). Bio-Sanitization Using Specific Bacteriophages to Control Escherichia coli O157:H7 in Cherry Tomatoes. Adv. J. Food Sci. Technol..

[19] Batalha L.S., Gontijo M.T.P., Teixeira A.V.N.D.C., Boggione D.M.G., Lopez M.E.S., Eller M.R., Mendonça R.C.S. (2021). Encapsulation in alginate-polymers improves stability and allows controlled release of the UFV-AREG1 bacteriophage. Food Res. Int..

[20] Young R., Wang I.-N., Roof W.D. (2000). Phages will out: Strategies of host cell lysis. Trends Microbiol..

[21] Matamp N., Bhat S.G. (2019). Phage Endolysins as Potential Antimicrobials against Multidrug Resistant Vibrio alginolyticus and Vibrio parahaemolyticus: Current Status of Research and Challenges Ahead. Microorganisms.

[22] Schmelcher M., Donovan D.M., Loessner M.J. (2012). Bacteriophage endolysins as novel antimicrobials. Future Microbiol..

[23] Young R. (2014). Phage lysis: Three steps, three choices, one outcome. J. Microbiol..

[24] Guo M., Feng C., Ren J., Zhuang X., Zhang Y., Zhu Y., Dong K., He P., Guo X.-K., Qin J. (2017). A Novel Antimicrobial Endolysin, LysPA26, against Pseudomonas aeruginosa. Front. Microbiol..

[25] Saier M.H., Reddy B.L. (2015). Holins in Bacteria, Eukaryotes, and Archaea: Multifunctional Xenologues with Potential Biotechnological and Biomedical Applications. J. Bacteriol..

[26] Young R. (2013). Phage lysis: Do we have the hole story yet?. Curr. Opin. Microbiol..

[27] Berry J.D., Rajaure M., Young R. (2013). Spanin function requires subunit homodimerization through intermolecular disulfide bonds. Mol. Microbiol..

[28] Gontijo M.T.P., Vidigal P.M.P., Lopez M.E.S., Brocchi M. (2021). Bacteriophages that infect Gram-negative bacteria as source of signal-arrest-release motif lysins. Res. Microbiol..

[29] Ghose C., Euler C.W. (2020). Gram-Negative Bacterial Lysins. Antibiotics.

[30] Love M.J., Abeysekera G.S., Muscroft-Taylor A.C., Billington C., Dobson R.C. (2020). On the catalytic mechanism of bacteriophage endolysins: Opportunities for engineering. Biochim. Biophys. Acta (BBA) Proteins Proteom..

[31] Oliveira H., Boas D.V., Emesnage S., Kluskens L.D., Lavigne R., Sillankorva S., Esecundo F., Eazeredo J. (2016). Structural and Enzymatic Characterization of ABgp46, a Novel Phage Endolysin with Broad Anti-Gram-Negative Bacterial Activity. Front. Microbiol..

[32] Altamirano F.L.G., Barr J.J. (2019). Phage Therapy in the Postantibiotic Era. Clin. Microbiol. Rev..

[33] Brives C., Pourraz J. (2020). Phage therapy as a potential solution in the fight against AMR: Obstacles and possible futures. Palgrave Commun..

[34] Kortright K.E., Chan B.K., Koff J.L., Turner P.E. (2019). Phage Therapy: A Renewed Approach to Combat Antibiotic-Resistant Bacteria. Cell Host Microbe.

[35] Principi N., Silvestri E., Esposito S. (2019). Advantages and limitations of bacteriophages for the treatment of bacterial infections. Front. Pharmacol..

[36] Kaur J., Kumar A., Kaur J. (2018). Strategies for optimization of heterologous protein expression in E. coli: Roadblocks and reinforcements. Int. J. Biol. Macromol..

[37] Rosano G.L., Ceccarelli E.A. (2014). Recombinant protein expression in Escherichia coli: Advances and challenges. Front. Microbiol..

[38] Nieuwkoop T., Claassens N.J., Van Der Oost J. (2018). Improved protein production and codon optimization analyses in Escherichia coli by bicistronic design. Microb. Biotechnol..

[39] Parret A.H., Besir H., Meijers R. (2016). Critical reflections on synthetic gene design for recombinant protein expression. Curr. Opin. Struct. Biol..

[40] Gopal G.J., Kumar A. (2013). Strategies for the Production of Recombinant Protein in *Escherichia coli*. Protein J..

[41] Jia B., Jeon C.O. (2016). High-throughput recombinant protein expression in *Escherichia coli*: Current status and future perspectives. Open Biol..

[42] Plotka M., Kaczorowska A.-K., Stefanska A., Morzywolek A., Fridjonsson O.H., Dunin-Horkawicz S., Kozlowski L., Hreggvidsson G.O., Kristjansson J.K., Dabrowski S. (2013). Novel Highly Thermostable Endolysin from Thermus scotoductus MAT2119 Bacteriophage Ph2119 with Amino Acid Sequence Similarity to Eukaryotic Peptidoglycan Recognition Proteins. Appl. Environ. Microbiol..

[43] Bailly-Bechet M., Danchin A., Iqbal M., Marsili M., Vergassola M. (2006). Codon Usage Domains over Bacterial Chromosomes. PLoS Comput. Biol..

[44] Chithambaram S., Prabhakaran R., Xia X. (2014). The effect of mutation and selection on codon adaptation in *Escherichia coli* bacteriophage. Genetics.

[45] Sharp P.M., Brenner S., Miller J.H. (2001). Codon Usage Bias. Encyclopedia of Genetics.

[46] Shilling P.J., Mirzadeh K., Cumming A.J., Widesheim M., Köck Z., Daley D.O. (2020). Improved designs for pET expression plasmids increase protein production yield in Escherichia coli. Commun. Biol..

[47] Kim S., Lee D.-W., Jin J.-S., Kim J. (2020). Antimicrobial activity of LysSS, a novel phage endolysin, against Acinetobacter baumannii and Pseudomonas aeruginosa. J. Glob. Antimicrob. Resist..

[48] Oliveira H., Thiagarajan V., Walmagh M., Sillankorva S., Lavigne R., Neves-Petersen M.T., Kluskens L., Azeredo J. (2014). A Thermostable Salmonella Phage Endolysin, Lys68, with Broad Bactericidal Properties against Gram-Negative Pathogens in Presence of Weak Acids. PLoS ONE.

[49] Khan F.M., Gondil V.S., Li C., Jiang M., Li J., Yu J., Wei H., Yang H. (2021). A Novel *Acinetobacter baumannii* Bacteriophage Endolysin LysAB54 With High Antibacterial Activity Against Multiple Gram-Negative Microbes. Front. Cell. Infect. Microbiol..

[50] Bai J., Yang E., Chang P.-S., Ryu S. (2019). Preparation and characterization of endolysin-containing liposomes and evaluation of their antimicrobial activities against gram-negative bacteria. Enzym. Microb. Technol..

[51] Lim J.-A., Shin H., Kang D.-H., Ryu S. (2012). Characterization of endolysin from a Salmonella Typhimurium-infecting bacteriophage SPN1S. Res. Microbiol..

[52] Lim J.-A., Shin H., Heu S., Ryu S. (2014). Exogenous Lytic Activity of SPN9CC Endolysin Against Gram-Negative Bacteria. J. Microbiol. Biotechnol..

[53] Yan G., Yang R., Fan K., Dong H., Gao C., Wang S., Yu L., Cheng Z., Lei L. (2019). External lysis of Escherichia coli by a bacteriophage endolysin modified with hydrophobic amino acids. AMB Express.

[54] Antonova N.P., Vasina D.V., Lendel A.M., Usachev E.V., Makarov V.V., Gintsburg A.L., Tkachuk A.P., Gushchin V.A. (2019). Broad Bactericidal Activity of the Myoviridae Bacteriophage Lysins LysAm24, LysECD7, and LysSi3 against Gram-Negative ESKAPE Pathogens. Viruses.

[55] Antonova N.P., Vasina D.V., Rubalsky E.O., Fursov M.V., Savinova A.S., Grigoriev I.V., Usachev E.V., Shevlyagina N.V., Zhukhovitsky V.G., Balabanyan V.U. (2020). Modulation of Endolysin LysECD7 Bactericidal Activity by Different Peptide Tag Fusion. Biomolecules.

[56] Blasco L., Ambroa A., Trastoy R., Bleriot I., Moscoso M., Fernandez-Garcia L., Perez-Nadales E., Fernández-Cuenca F., Torre-Cisneros J., Oteo-Iglesias J. (2020). In vitro and in vivo efficacy of combinations of colistin and different endolysins against clinical strains of multi-drug resistant pathogens. Sci. Rep..

[57] Ramos C., Abreu P., Nascimento A., Ho P. (2004). A high-copy T7 Escherichia coli expression vector for the production of recombinant proteins with a minimal N-terminal His-tagged fusion peptide. Braz. J. Med Biol. Res..

[58] Yang P.-C., Liu Z.-Q. (2012). Construction of pET-32 α (+) vector for protein expression and purification. N. Am. J. Med. Sci..

[59] Bornhorst J.A., Falke J.J. (2000). Purification of proteins using polyhistidine affinity tags. Methods Enzymol..

[60] Mondal S.I., Draper L.A., Ross R.P., Hill C. (2020). Bacteriophage endolysins as a potential weapon to combat Clostridioides difficile infection. Gut Microbes.

[61] Briers Y., Walmagh M., Grymonprez B., Biebl M., Pirnay J.-P., Defraine V., Michiels J., Cenens W., Aertsen A., Miller S. (2014). Art-175 Is a Highly Efficient Antibacterial against Multidrug-Resistant Strains and Persisters of *Pseudomonas aeruginosa*. Antimicrob. Agents Chemother..

[62] Gutiérrez D., Fernández L., Rodríguez A., García P. (2018). Are Phage Lytic Proteins the Secret Weapon to Kill *Staphylococcus aureus*?. mBio.

[63] Loeffler J.M., Nelson D., Fischetti V.A. (2001). Rapid Killing of *Streptococcus pneumoniae* with a Bacteriophage Cell Wall Hydrolase. Science.

[64] Gilmer D.B., Schmitz J.E., Euler C.W., Fischetti V.A. (2013). Novel Bacteriophage Lysin with Broad Lytic Activity Protects against Mixed Infection by Streptococcus pyogenes and Methicillin-Resistant Staphylococcus aureus. Antimicrob. Agents Chemother..

[65] Pastagia M., Euler C., Chahales P., Fuentes-Duculan J., Krueger J.G., Fischetti V.A. (2010). A Novel Chimeric Lysin Shows Superiority to Mupirocin for Skin Decolonization of Methicillin-Resistant and -Sensitive Staphylococcus aureus Strains. Antimicrob. Agents Chemother..

[66] Gerstmans H., Rodriguez-Rubio L., Lavigne R., Briers Y. (2016). From endolysins to Artilysin^®^s: Novel enzyme-based approaches to kill drug-resistant bacteria. Biochem. Soc. Trans..

[67] Fischetti V.A. (2010). Bacteriophage endolysins: A novel anti-infective to control Gram-positive pathogens. Int. J. Med. Microbiol..

[68] Fischetti V.A. (2006). Using phage Lytic Enzymes to Control Pathogenic Bacteria. BMC Oral Health.

[69] Nelson D., Loomis L., Fischetti V.A. (2001). Prevention and elimination of upper respiratory colonization of mice by group A streptococci by using a bacteriophage lytic enzyme. Proc. Natl. Acad. Sci. USA.

[70] Gondil V.S., Harjai K., Chhibber S. (2020). Endolysins as emerging alternative therapeutic agents to counter drug-resistant infections. Int. J. Antimicrob. Agents.

[71] Ciepluch K., Skrzyniarz K., Barrios-Gumiel A., Quintana S., Sánchez-Nieves J., De La Mata F.J., Maciejewska B., Drulis-Kawa Z., Arabski M. (2019). Dendronized Silver Nanoparticles as Bacterial Membrane Permeabilizers and Their Interactions with *P aeruginosa* Lipopolysaccharides, Lysozymes, and Phage-Derived Endolysins. Front. Microbiol..

[72] Finnegan S., Percival S. (2015). EDTA: An Antimicrobial and Antibiofilm Agent for Use in Wound Care. Adv. Wound Care.

[73] Bollenbach T. (2015). Antimicrobial interactions: Mechanisms and implications for drug discovery and resistance evolution. Curr. Opin. Microbiol..

[74] Sercombe L., Veerati T., Moheimani F., Wu S., Sood A.K., Hua S. (2015). Advances and Challenges of Liposome Assisted Drug Delivery. Front. Pharmacol..

[75] Zampara A., Sørensen M.C.H., Grimon D., Antenucci F., Vitt A.R., Bortolaia V., Briers Y., Brøndsted L. (2020). Exploiting phage receptor binding proteins to enable endolysins to kill Gram-negative bacteria. Sci. Rep..

[76] Lukacik P., Barnard T.J., Keller P.W., Chaturvedi K.S., Seddiki N., Fairman J.W., Noinaj N., Kirby T.L., Henderson J.P., Steven A.C. (2012). Structural engineering of a phage lysin that targets Gram-negative pathogens. Proc. Natl. Acad. Sci. USA.

[77] Briers Y., Walmagh M., Van Puyenbroeck V., Cornelissen A., Cenens W., Aertsen A., Oliveira H., Azeredo J., Verween G., Pirnay J.-P. (2014). Engineered Endolysin-Based “Artilysins” To Combat Multidrug-Resistant Gram-Negative Pathogens. mBio.

[78] Mikoulinskaia G.V., Chernyshov S.V., Shavrina M.S., Molochkov N.V., Lysanskaya V.Y., Zimin A.A. (2018). Two novel thermally resistant endolysins encoded by pseudo T-even bacteriophages RB43 and RB49. J. Gen. Virol..

[79] Plotka M., Kaczorowska A.-K., Morzywolek A., Makowska J., Kozlowski L., Thorisdottir A., Skírnisdottir S., Hjörleifsdottir S., Fridjonsson O.H., Hreggvidsson G.O. (2015). Biochemical Characterization and Validation of a Catalytic Site of a Highly Thermostable Ts2631 Endolysin from the *Thermus scotoductus* Phage vB_Tsc2631. PLoS ONE.

[80] Plotka M., Kapusta M., Dorawa S., Kaczorowska A.-K., Kaczorowski T. (2019). Ts2631 Endolysin from the Extremophilic *Thermus scotoductus* Bacteriophage vB_Tsc2631 as an Antimicrobial Agent against Gram-Negative Multidrug-Resistant Bacteria. Viruses.

[81] Wang F., Xiong Y., Xiao Y., Han J., Deng X., Lin L. (2020). MMPphg from the thermophilic *Meiothermus* bacteriophage MMP17 as a potential antimicrobial agent against both Gram-negative and Gram-positive bacteria. Virol. J..

[82] Knecht L.E., Veljkovic M., Fieseler L. (2020). Diversity and Function of Phage Encoded Depolymerases. Front. Microbiol..

[83] Wang C., Li P., Zhu Y., Huang Y., Gao M., Yuan X., Niu W., Liu H., Fan H., Qin Y. (2020). Identification of a Novel *Acinetobacter baumannii* Phage-Derived Depolymerase and Its Therapeutic Application in Mice. Front. Microbiol..

[84] Pan Y.-J., Lin T.-L., Lin Y.-T., Su P.-A., Chen C.-T., Hsieh P.-F., Hsu C.-R., Chen C.-C., Hsieh Y.-C., Wang J.-T. (2015). Identification of Capsular Types in Carbapenem-Resistant Klebsiella pneumoniae Strains bywzcSequencing and Implications for Capsule Depolymerase Treatment. Antimicrob. Agents Chemother..

[85] Fernández-Ruiz I., Coutinho F.H., Rodriguez-Valera F. (2018). Thousands of Novel Endolysins Discovered in Uncultured Phage Genomes. Front. Microbiol..

[86] Gondil V.S., Chhibber S. (2021). Bacteriophage and Endolysin Encapsulation Systems: A Promising Strategy to Improve Therapeutic Outcomes. Front. Pharmacol..

[87] Barreto P.D.S., Vellas B., Rolland Y. (2021). Physical activity and exercise in the context of SARS-CoV-2: A perspective from geroscience field. Ageing Res. Rev..

[88] Agu R.U., Ugwoke M.I., Armand M., Kinget R., Verbeke N. (2001). The lung as a route for systemic delivery of therapeutic proteins and peptides. Respir. Res..

[89] Murray E., Draper L., Ross R., Hill C. (2021). The Advantages and Challenges of Using Endolysins in a Clinical Setting. Viruses.

[90] Furfaro L.L., Payne M.S., Chang B.J. (2018). Bacteriophage Therapy: Clinical Trials and Regulatory Hurdles. Front. Cell. Infect. Microbiol..

[91] Love M.J., Bhandari D., Dobson R.C.J., Billington C. (2018). Potential for Bacteriophage Endolysins to Supplement or Replace Antibiotics in Food Production and Clinical Care. Antibiotics.

